# The Plasticity of Our Fears: Affective Politics in the European Migration Crisis

**DOI:** 10.1007/s12115-021-00643-2

**Published:** 2021-10-25

**Authors:** Volker M. Heins

**Affiliations:** Institute for Advanced Study in the Humanities, University of Duisburg-Essen, Goethestrasse 31, 45128 Essen, Germany

**Keywords:** Affective politics, Fear, Heinrich Popitz, Migration crisis, Refugees, Rhetoric of reaction

## Abstract

In the field of migration politics, a dominant rhetoric argues that liberal immigration and asylum policies must be avoided because they will inevitably lead to anti-immigration backlashes that exacerbate the very conditions they were supposed to remedy. Drawing on the work of German sociologist Heinrich Popitz and empirical data on the aftereffects of the European migration crisis, the article criticizes this “rhetoric of reaction” (Albert Hirschman) for ignoring the many variables shaping the consequences of more open borders. Backlashes to immigration are real and pose a constraint for liberal immigration policies, but these backlashes are not necessarily politically successful. Societies react neither uniformly nor automatically to rising immigration. A critical variable is the fear engendered by the (real, expected, or imagined) arrival of large numbers of migrants, and this fear can be either ramped up to paranoid levels or calmed by a politics of hope aimed at restoring what Popitz called the “human openness to the world.”

## A Rhetoric of Reaction

In April 2021, New York Times columnist Bret Stephens (2021) wrote an opinion piece in which he called on US President Joe Biden to “complete the wall” between the USA and Mexico in order to stop illegal border crossings. Without the wall, he argued, “the United States risks a version of the European migration crisis of 2015. That’s the one that contributed heavily to the Brexit vote, turbocharged the rise of far-right parties like France’s National Front and the Alternative for Germany, and paved the way to Trump’s election.” Stephens rejects the short-lived open doors policy of some European Union member states such as Germany and Sweden toward desperate migrants from the Middle East and elsewhere, not because he thinks migrants are not worthy of our “compassion and respect,” but rather because he predicts that open borders will lead to a surge of anti-immigrant sentiment, xenophobia, and racism. Only the wall, he concludes, will protect us “against the next populist revolt, which is sure to overtake our politics” (Stephens 2021) if no wall is built. By claiming that more open borders will *inevitably*, and contrary to the intentions of pro-immigrant activists, make life harder for migrants, the journalist offers a textbook example of what Albert Hirschman called the “rhetoric of reaction.” That even Stephens, widely viewed as a moderate conservative, draws on the rhetoric of reaction is a sign of how pervasive it is. A prominent mode of this rhetoric is based on the claim that liberal or progressive policies must be avoided because they will, predictably and perversely, lead to backlashes that exacerbate the very conditions they were supposed to remedy (Hirschman 1991, Chap. 2).

The problem is that the rhetoric of reaction grossly simplifies complex causal dynamics by assuming that progressive political action will produce not only unintended consequences, but also the exact opposite of what was intended. However, as Hirschman writes, “the perverse effect is by no means the only conceivable variety of unintended consequences and side effects” (Hirschman 1991, p. 38). While it is true that a sudden spike in immigration often leads to anti-immigration backlashes, there is no automatic causality linking the two phenomena, certainly not in the long run. The rhetoric of reaction ignores the many variables shaping the consequences of liberal immigration and asylum policies. This is the central claim I wish to defend in this short paper.

There is no doubt that the arrival of high numbers of asylum seekers or other migrants within a short period of time provides ample opportunities for a far-right politics of fear. However, there is no assurance that a strategy of fearmongering will actually work. A politics of fear is always possible, but so is a politics of hope. For example, Stephens is right in pointing out that the far-right party Alternative for Germany (AfD) benefited from the refugee situation in Germany, but he forgets to mention that, if we take a longer-term perspective, the pro-migrant Green Party and other moderate forces have been much more successful.^1^ This can be generalized for almost all European countries. Survey data from 13 EU member states show that the migration crisis has not changed the overall trend toward *growing* acceptance of immigration over the last two decades, including immigration from outside of Europe (Stöhr 2018; Dennison and Geddes 2019).

Whether a politics of fear prevails over a politics of hope depends to a large extent on the affective politics of multiple actors in society. By affective politics I mean all activities aimed at shaping and transforming fleeting moods into more stable emotions, beliefs, or ideologies that anchor people in particular identities, beliefs, and practices. Affects are “vital” or “visceral” forces or energies that attract us to, or repel us from, bodies, objects, thoughts, or situations. Once stabilized by narrative and interpretation, affect makes sure that we remain invested in certain behaviors, goals, facts, and stories because we feel that they intensely matter to us. Only because we are affective beings are we able to feel our actions or inactions in ways that are either meaningful and satisfying or pointless and frustrating. Unlike personal sentiment, which is already fixed through language and experience, affect is not yet fully formed and shaped. Fear is a good example. The fear for which we find words is already disappearing from our hearts. Fear is also a good example for the fluid and viral nature of affects which supports the remixing of fact and fiction, reality, and fantasy (Grossberg 1992; Seigworth and Gregg 2010; Papacharissi 2015).

If all human activity is driven by desire and pain, hope, and fear, then politics, too, always has an affective dimension. Max Weber’s rigid opposition between a “politics of emotion” and a “politics of reason” (Weber 1994, p. 124), and his preference for the latter, is therefore mistaken. There is no pure reason independent of our capacity to be affected and touched by events, people, and situations. From Weber onward, affectivity and emotion have been relegated to irrational masses as opposed to enlightened elites, or to the politics of the “street” as opposed to the politics of parliaments and rational political parties. But these oppositions are false.

To overcome these false oppositions, I draw on the work of German sociologist Heinrich Popitz whose book *Phenomena of Power* was discovered only a few years ago in the English-speaking world.^2^ According to Popitz, all politics is affective in the sense that politics always entails struggles over the fears and hopes of citizens, residents, and aliens. Fear, like hope, is not a natural or automatic reaction to events but rather a malleable affect always shaped by power relations. This is what Popitz calls the “social plasticity” of our feelings (Popitz 2017, p. 67). Political struggles over affect are driven by “instrumental power,” which he largely conceives in terms of fear-inducing threats. Popitz gives analytic primacy to fear, violence, and the infliction of harm as tools of power. States and other powerful actors “make use of the plasticity of the fears and hopes” (Popitz 2017, p. 67) by engaging in affective politics. Recent mass migrations, which have been constructed and represented as “crises,” offer valuable insights into the workings of affective politics and the changeability of the hopes and fears of both natives and migrants.

## Has There Really Been a Migration Crisis?

If by “crisis” we understand a dangerous yet decisive turning point, there was no such thing as a migration crisis in Europe in 2015. It is not the case that those who arrived exceeded the absorptive capacities of labor markets, the educational system, or the welfare state by any objective measure. Nor did the popular willingness to receive migrants disappear or reach a breaking point. Similarly, when we look at migration policies of the EU and its member states, there was shock and bewilderment among the elites, but no crisis understood as an opportunity to change course. No effort was made by governments to explore the “decolonial” possibilities of (re-)opening legal migration routes from Africa and the Middle East to Europe and of creating new infrastructures and “logistics” of migration (Achiume 2019; Mezzadra 2019). Instead, European states continued to bribe and pressure authoritarian states and rogue militias to halt migrants long before they reach Europe. Laws against human smuggling were increasingly used to prosecute doctors, landlords, or ordinary citizens who provided medical and legal assistance, food, shelter, or other forms of support to undocumented migrants (Ben-Arieh and Heins 2021; Schack and Witcher 2021). Italy and Malta prohibited civilian search and rescue ships from docking in their ports. Greece, Croatia, and other states engaged in illegal pushbacks of migrants, some of whom were picked up—or kidnapped (Tazzioli and De Genova 2020) —in the Mediterranean and sent back to Libya where they were subject to human rights violations including torture, rape, indefinite arbitrary detention, killings, human trafficking, and forced labor (Amnesty International 2021; Border Violence Monitoring Network 2021). All of this was meant to “upgrade threats into a power to frighten others” (Popitz 2017, p. 13) — yet with limited success.

European governments were unable or unwilling to pause and reflect on the justice and long-term feasibility of militarized border controls. Policy inertia prevailed over political creativity and any change of policy. There was no turning point and hence no crisis, only a lot more of the same (Guiraudon 2018; Scheel 2018; Landau 2019). The original medical meaning of the word “crisis” refers to the point in an illness at which the patient either dies or recovers. In this sense, only migrants suffered a crisis. For them, it was often a matter of life or death whether they would make it to Europe’s edges or perish along the way. Unlike governments, refugees, who were believed to be without power and agency, exhibited surprising initiative and imagination. At least for a moment, they disrupted the political order of things. It is estimated that 80% of the migrants arriving in Europe in 2015 were originally from Afghanistan, Iraq, and Syria—all countries ravaged by the disastrous post-9/11 interventions organized by the USA and supported by changing coalitions of various EU member states. In each of these instances of senseless bombing and fighting, new refugee flows were created, composed of growing numbers of individuals determined to overcome international border regimes designed to keep them where they are. The exiled Syrian writer Yassin al-Haj Saleh has made the point that the refugees and migrants from Afghanistan, the Middle East, or Africa who have sought shelter in Europe “have invented something new in international politics: crossing multiple borders, that is, erasing borders in an unprecedented way that can be imitated by others” (Al-Haj Saleh 2018).

Although the term “crisis” is generally used in a misleading way when applied to the refugee situation in Europe and its geopolitical neighborhood, I would still maintain that there is a precise sociological sense in which we can indeed speak of a European migration crisis. The situation created by the sudden arrival of more than a million refugees from outside of Europe aroused and moved the whole of European society, even though people were not moved uniformly in one direction. Citizens were deeply divided over the very meaning of “Europe” and unsure what to fear and what to hope for. In this sense, Europe was and is still going through what Jeffrey Alexander, writing in a different context, has called a “deep crisis” that puts at stake the “symbolic, sacred center of society” (Alexander 2003, p. 157). The depth of the crisis was not due to the mere fact of large numbers of migrants arriving from across the Mediterranean Sea or overland through Southeast Europe. Rather, the movement of migrants gave rise to fundamental questions of who “we Europeans” are, how we want to be seen by the outside world, and what kind of society we wish to become. These still unanswered questions have opened a period of intense social drama.

In this way, the arrival of unprecedented numbers of irregular border crossers from the Global South laid bare and exacerbated longstanding tensions not only within each European society, but also between northern and southern, western, and eastern member states. As a consequence, the already “faltering project” (Habermas 2009; Balibar 2016) of European unification was further undermined. Europe may possess a symbolic center, enshrined in founding documents and declarations, but this center turned out to be empty—or so it seemed to many Europeans. This disillusionment was the result of the expansion of the scope of the debate about migration issues. No longer was the debate confined to experts and bureaucrats. It now included populist leaders and their followers, leftist academics, mayors across Europe, NGOs and No Borders activists, and of course migrants themselves. Throughout society, people held strong views on what was happening before their eyes. All European countries were divided (though not evenly) between those who saw the admission of the refugees as a catastrophic deviation from the most sacred values of Europe, and those who felt that they were witnessing a big step forward toward a more open, truly postcolonial European Union.

## Anti-Migrant Affective Epidemics

Crucial for the development of anti-immigration attitudes is, among other things, the perception of threats and fear of outsiders. Anti-immigrant movements appeal to fear and, consequently, to the need to take protective action against those who cause fear. Typically, the protective action called for is a preemptive action targeting sources of threat that have not yet materialized. The dreaded “Islamization” or, more recently, “Africanization” of Europe is allegedly already underway, but there is still time to act against what one day might become a pervasive reality.

An effective anti-immigration movement can only emerge if a critical mass of people genuinely feels that the world beyond the borders of their own countries is full of dark and dangerous places. For this reason, the inhabitants of those places need to be stripped of their freedom of movement to make sure that they stay where they are. Fear is generated by superimposing preconceived negative ideas about certain stigmatized groups (Muslims, Jews, blacks, etc.) onto actual persons who in the process morph into “others,” radically different from “us.” Sometimes hostile stereotypes are developed into horrifying narratives about the destruction of white, Western civilization as a result of mass immigration from the Global South. A prominent example is *The Camp of the Saints*, a 1973 French dystopian fiction novel by Jean Raspail, which in 2011 returned to bestseller lists. Raspail’s influential novel is based on the “Apocalypse of John” in the *Book of Revelation* that prophesizes the arrival of evil tribes who will destroy the world in the last moments of history. Raspail transforms this vision into a more mundane scenario in which hordes of faceless, half-naked, and only half-human migrants from Asia arrive upon the shores of the French Riviera to destroy “us.” The alternative suggested in the deeply racist novel is to destroy “them.” The book was translated into numerous languages and publicly endorsed by various far-right figures in Europe and by people close to the Trump administration, including politicians such as former Republican Congressman Steve King (Stieber 2019).

It is thus through the workings of a specific kind of affective politics that citizens are made fearful of migrants. This is a depressingly familiar narrative throughout global migration history: people claim and feel that there are *too many* migrants, who are *too different* from the culture of the receiving country.^3^ Fearful subjects tend to picture migrants not as individuals with rights, but as dark, unwashed masses advancing like a flood. Biblical images of immigrants threatening to “deluge” our towns and cities were common in the USA in the 1920s as well as in Europe. Max Weber, for example, likened Polish seasonal workers to “swarms of nomads from the east” descending upon Germany (cited in Mayer 1955, p. 34). Such deeply affective images were accompanied by fantasies or plans of building impenetrable walls or dykes, “closing the gates” to outsiders, and erecting a “Great Barrier” against the mass influx of Asians, East Europeans, or other peoples deemed less civilized (Bashford 2014, pp. 123–124).

Given these carefully cultivated fears of the coming inundation of civilized countries by itinerant barbarians, it is not surprising that recent refugee movements across the Western Balkans and the Mediterranean Sea toward Europe struck terror into the hearts of many. Nor is it surprising that a politics of fear could flourish even in places where migrants failed to arrive. For fear to arise, it was enough that many Europeans were susceptible to *imagining* large-scale arrivals of migrants on their shores, and that this imagination was charged with great fear. The refugee movement was constructed not only as a real “crisis,” but also as a matter of affect—something that *felt* real even in places where it did not materialize (yet).

As a matter of affect, the imaginary dangers of migration can easily explode into what Lawrence Grossberg once called an “affective epidemic.” Migration and, more specifically, irregular migration have been invested with an excess of meaning. Like other phenomena before (such as “drugs” or “dysfunctional families”), many now see migration everywhere, as “the new universal culprit” (Grossberg 1992, p. 284) for all kinds of social ills. When the emotional concern with the consequences of migration spirals out of control, nothing else seems to matter and all things seem to be somehow connected to migration. As former German Interior Minister Horst Seehofer declared a few years ago: migration is the “mother of all problems” (cited in Young 2018).

Statements like this have set the emotional tone for many European conservatives and the new far right. Divisions emerge over the question of who created the problem of mass immigration, and how the problem can be fixed. The question has been answered by blaming allegedly “weak” governments and civil society initiatives, criminal people smugglers, or individual figures such as the Jewish billionaire Georges Soros, who in 2017 was portrayed by the Hungarian government in a nationwide poster campaign as a hook-nosed, maliciously grinning conspirator plotting to destroy Europe by flooding the continent with Muslim migrants. According to Hanno Loewy, director of the Jewish Museum of Hohenems in Austria, this was “the most effective anti-Semitic campaign of the present age”—a campaign that skillfully combined Islamophobia with anti-Semitism (Loewy 2020).

Popitz writes that those who are in power “can teach others to fear” by conjuring up or inflating threats while exploiting “the generic social plasticity of the human being” (Popitz 2017, p. 67). But those who wrongly think of themselves as powerless, for instance white males who feel oppressed and silenced by increasingly vocal ethnic minorities, can also teach each other to be fearful. This is achieved by forming new affective publics, especially through the use of digitally networked media (Papacharissi 2015). Fear is generated and made contagious by establishing an interpretive framework that encourages members of the public to read every scuffle between migrant and native youths, every woman wearing a hijab, every crime committed by an asylum seeker as an ominous *sign* of an ongoing or imminent clash of cultures. Teaching people to interpret events in such a way includes teaching them new feeling rules. They are told that they ought to get angry, and show their anger, if politicians do not get tough on smugglers and humanitarian NGOs, or if someone shows mercy to irregular migrants. They are also told who deserves recognition, for example politicians such as Matteo Salvini, a former member of the Italian government, who—perhaps alluding to the aforementioned novel by Jean Raspail—repeatedly called African migrants mere *carne umana*, “human flesh.” In a similar tone, participants in one of the numerous rightwing demonstrations in Dresden, upon hearing about humanitarian efforts to rescue migrants in the Mediterranean, chanted the slogan *Absaufen, absaufen!*, “Let them drown!” (cited in Heins 2021, pp. 35–36, 149).

These instances of public celebration of callousness and cruelty are but symptoms of the evolution of initially shapeless anti-immigrant feelings into a new and robust affective constellation that now governs the relations between sections of Europe’s native population and migrants, many of whom are kept in detention centers in Greece or other hotspots, or risk their lives on their way to Europe. This new affective constellation makes sure that the suffering, neglect, and death of migrants is met neither with empathy nor the urge to honor international legal obligations laid down in the Geneva Refugee Convention or the Convention on the Law of the Sea. For now, ethnocentric visions of conservatism motivate only a few “to act so as to inflict harm” (Popitz 2017, p. 17) on migrants and refugees. Instead of becoming active perpetrators, most individuals raised within the new culture of callousness are content to remain bystanders to the misery inflicted on others by policies and actions that perpetuate harm.

## Human Openness to the World

I have argued that the rhetoric of reaction, which claims that liberal immigration and asylum policies are bound to produce their own backlash, ignores the many variables shaping the consequences of more open borders. The social world is not governed by iron laws, and the future is much less predictable than the rhetoric of reaction insinuates. Backlashes to immigration are real and pose a constraint on more open borders, but these backlashes are not necessarily politically successful. Societies react neither uniformly nor automatically to rising immigration (Del Savio 2020). One critical variable is the fear engendered by the (real, expected, or imagined) arrival of large numbers of migrants, in particular if these migrants come from unknown (or, in the case of former colonies, no longer well known) places including war zones. This fear is not misplaced or irrational per se. However, as Popitz has shown, fear can be deliberately ramped up to paranoid levels to influence the conduct of populations.

A certain level of fearfulness is, of course, always reasonable. As Hobbes has argued, without fear, humans would fall prey to all kinds of dangers, including dangers such as robbery, violence, rape, and murder. One of Hobbes’ great insights is that not all humans are evil and dangerous, but some are, and “we cannot tell the good and the bad apart” (Hobbes 1998, p. 11) before getting to know them. What Hobbes did not foresee is the emergence of modern racism and other affective ideologies. These ideologies wrongly suggest that there is indeed an a priori way to tell good and bad people apart. It is this promise of predictability and simplicity that makes racism and similar ideologies so attractive to so many. Racism, anti-Semitism, Islamophobia, and similar irrational and exclusionary attitudes are based on the false belief that markers such as language, skin color, religious belief, or clothing are signs by which one can recognize the wrong kind of people. Affective ideologies make use of the plasticity of our fears by molding them in such a way that it feels like the social world is divided into “us versus them.” As a result, fear is both contained and inflated beyond proportion. Fear is contained when people are made to believe that all danger comes from an ideologically “known” group outside of society. At the same time, these outsiders are often portrayed in apocalyptic terms, causing a xenophobic fear that outstrips its ostensible causes, unlike Hobbes’ reasonable fear.

A politics of hope aimed at undermining the prevalent hierarchical and racialized understanding of rights to mobility needs to address and counter the affective forces fueling anti-immigration backlashes. At the most fundamental level, this is possible, because all humans, resident populations as well as migrants, are capable of adapting to new circumstances. As Popitz writes, “we can be sure that the experience of being compelled to adapt and of being able to adapt is as ancient as the story of the human being. It travelled alongside every stream of refugees […] Ultimately this ability to adapt to new situations is an expression of the ‘human openness to the world,’ our capacity of arranging ourselves for more than one world” (Popitz 2017, pp. 68–69). Ethnocentric conservatism including its more extreme, neo-fascist versions—as well as analogous affective formations such as, for example, Islamic fundamentalism—are an attack on this characteristic ability of humans to live in more than one world and adapt to new situations.

The desire of retreating into one world only cannot be overcome by inviting fearful xenophobes to publicly participate in a different mode of moral reasoning. Instead, from experiences in places that have welcomed many new migrants and refugees in recent years, we can draw three lessons on how to counter xenophobic backlashes. First, we know from empirical research that, on average, far-right parties did not perform well in municipalities that have combined the hosting of refugees with efforts to facilitate interaction between locals and newly arrived migrants. This is true even in countries that are not known for being particularly migrant friendly (Steinmayr 2017). The “signs” by which racists claim to tell good and bad people apart at a glance can be destabilized and made meaningless. People can unlearn to be fearful and restore their openness to the world.

Second, anti-immigration movements have been bogged down wherever it was possible to change the subject of the local or national conversation. Germany, for example, now has the fifth highest population of refugees in the world (UNHCR 2021), with 1.7 million people having applied for asylum between 2015 and 2019, but in the run-up to the 2021 federal election, climate change and the COVID-19 pandemic replaced immigration as the most salient issues. As a result of a successful “struggle to change what matters” (Grossberg 1992, p. 281), people’s affective relations with the world were redirected. It has become increasingly difficult to convince the public that migration is at the root of all problems.

Finally, not only refugees and migrants are vulnerable to power. Xenophobic movements and political parties, too, can be made to acquiesce to the new realities created by mass immigration. Their “budgets of fear and hope” (Popitz 2017, p. 68) can be influenced by powerful pro-migrant social alliances including governments, either by calming their fears or, more auspiciously, by dashing their hopes of rebuilding a closed society for whites only.

## Footnotes

1 In all major German cities that have welcomed and hosted the bulk of refugees arriving in or after 2015—Munich, Frankfurt, Cologne, Dortmund, and others—the Green Party has become either the most popular or the second-most popular party in recent local elections. In the recent federal elections, Social Democrats and the Green Party have grown disproportionately compared to the far-right Alternative for Germany which has lost throughout the country except in some of the eastern states. It is also worth emphasizing that throughout Europe, numerous cities—Barcelona, Ljubljana, Naples, Zurich, and many others—publicly declared their intention to become “cities of refuge” (Christoph and Kron 2019).

2 John A. Hall has called the book a “classic” and “one of the most important texts ever written about power” (Hall 2017, p. 357).

3 It is not that high numbers of migrants give rise to public fear, but the other way round. First there is fear, and then fear produces fantasies of ever-growing numbers. Moreover, entrenched ideas of unbridgeable otherness can lead to situations in which even very small numbers of migrants cause panic. In 1870, only 88 Chinese workers were counted in San Francisco. Still, there were big demonstrations and mob attacks on Chinese people who were seen as being too different from Californians, and thus too many (Sandmeyer 1991, pp. 22, 47–48).
